# Ammonia in the Bites of Venomous Snakes

**Published:** 1828-04

**Authors:** 


5. *Ammonia in the bites of venemous snakes.* The treatment of these affections is exceedingly empyrical. In the number of the Journal just quoted, Dr. Moore, of Mississippi, has augmented the mass of evidence in favor of ammonia, by the publication of three cases successfully treated with that medicine. Within the last twenty years, he has seen fourteen other persons cured by the same remedy. They were all bitten by Rattle-snakes, or other serpents known to be venemous. He gives the strong liquor ammoniæ in tea spoonful doses, internally, at short intervals; and applies it also to the wound.

We were lately required to treat the bite of a *copperhead*, one of our most poisonous snakes, which inserted its two fangs in the instep of a young woman. Under the combined influence of cupping and ammoniated alcohol, she speedily recocovered.

